# Pre-treatment assessment of chemotherapy for cancer patients: a multi-site evidence implementation project of 74 hospitals in China

**DOI:** 10.1186/s12912-024-01997-8

**Published:** 2024-05-11

**Authors:** Jie Lai, Bianca Pilla, Matthew Stephenson, Alison Brettle, Chunlan Zhou, Wenji Li, Chaixiu Li, Jiaqi Fu, Shisi Deng, Yujie Zhang, Zihan Guo, Yanni Wu

**Affiliations:** 1 Nanfang Hospital, Southern Medical University, Guangzhou, PR China; 2 School of Nursing, Southern Medical University, Guangzhou, PR China; 3 JBI, School of Public Health, University of Adelaide, Adelaide, Australia; 4 School of Health & Society, University of Salford, Manchester, UK

**Keywords:** Evidence-based practice, Clinical audit, Quality improvement, Cancer patients, Chemotherapy, Nursing assessment

## Abstract

**Background:**

Chemotherapy, whilst treating tumours, can also lead to numerous adverse reactions such as nausea and vomiting, fatigue and kidney toxicity, threatening the physical and mental health of patients. Simultaneously, misuse of chemotherapeutic drugs can seriously endanger patients' lives. Therefore, to maintain the safety of chemotherapy for cancer patients and to reduce the incidence of adverse reactions to chemotherapy, many guidelines state that a comprehensive assessment of the cancer patient should be conducted and documented before chemotherapy. This recommended procedure, however, has yet to be extensively embraced in Chinese hospitals. As such, this study aimed to standardise the content of pre-chemotherapy assessment for cancer patients in hospitals and to improve nurses' adherence to pre-chemotherapy assessment of cancer patients by conducting a national multi-site evidence implementation in China, hence protecting the safety of cancer patients undergoing chemotherapy and reducing the incidence of adverse reactions to chemotherapy in patients.

**Methods:**

The national multi-site evidence implementation project was launched by a JBI Centre of Excellence in China and conducted using the JBI approach to evidence implementation. A pre- and post-audit approach was used to evaluate the effectiveness of the project. This project had seven phases: training, planning, baseline audit, evidence implementation, two rounds of follow-up audits (3 and 9 months after evidence implementation, respectively) and sustainability assessment. A live online broadcast allowed all participating hospitals to come together to provide a summary and feedback on the implementation of the project.

**Results:**

Seventy-four hospitals from 32 cities in China participated in the project, four withdrew during the project's implementation, and 70 hospitals completed the project. The pre-and post-audit showed a significant improvement in the compliance rate of nurses performing pre-chemotherapy assessments for cancer patients. Patient satisfaction and chemotherapy safety were also improved through the project's implementation, and the participating nurses' enthusiasm and belief in implementing evidence into practice was increased.

**Conclusion:**

The study demonstrated the feasibility of academic centres working with hospitals to promote the dissemination of evidence in clinical practice to accelerate knowledge translation. Further research is needed on the effectiveness of cross-regional and cross-organisational collaborations to facilitate evidence dissemination.

**Supplementary Information:**

The online version contains supplementary material available at 10.1186/s12912-024-01997-8.

## Background

Cancer incidence and mortality rates remain on the rise on a global scale, and cancer is the leading cause of death in every country [1]. Meanwhile, according to the World Health Organization’s forecasts for 2019, cancer is the first or second leading cause of death before age 70 in 112 out of 183 countries [2]. Consequently, there is a need to strengthen investment in cancer healthcare to improve cancer survival rates [3]. Chemotherapy is routinely used as one of the foremost cancer treatments and to reduce the risk of cancer recurrence [4]. Although chemotherapy has contributed significantly to the treatment of cancer, it also has various adverse effects that substantially affect cancer patients' outcomes and quality of life [5]. As such, ensuring the safety of chemotherapy for cancer patients and reducing the incidence of adverse reactions due to chemotherapy is an essential concern for healthcare professionals.

Studies have shown that an accurate and comprehensive assessment of cancer patients before chemotherapy can prevent and reduce chemotherapy complications and optimise chemotherapy outcomes [6, 7]. Furthermore, the 2016 update of the American Society of Clinical Oncology/Oncology Nursing Society Safety Standards for Chemotherapy states that the nursing record sheet should include nursing assessment and documentation of all indicators of chemotherapy for cancer patients [8]. In 2017, the Competencies and Standards for Cancer Chemotherapy Nursing Practice, published by the Canadian Association of Oncology Nurses, also clearly stated that nursing staff should conduct and document a thorough assessment of cancer patients before and after chemotherapy [9]. Although pre-chemotherapy assessment of cancer patients has also attracted the attention of professionals in China, awareness and practice of pre-chemotherapy assessment of cancer patients by clinical nurses in China still need to be improved [10]. Moreover, as practical guidelines for the nursing assessment of chemotherapy patients in China have yet to be developed, there is no standard practice for assessing cancer patients undergoing chemotherapy [10]. It is, therefore, imperative to address gaps in knowledge and practice, improve the quality of care and preserve the safety of chemotherapy for cancer patients.

It is widely recognised that evidence-based practice (EBP) is an essential mechanism for improving the quality of healthcare delivery [11], with numerous studies advocating for nurses' involvement in EBP as an essential method for providing high-quality safe care, improved patient outcomes, and reduced costs [12, 13]. Previous studies have found, however, that they are not well prepared for EBP, and although they have a positive attitude towards EBP, they lack the capacity and confidence to implement it [14–16]. This is true for nurses in China who lack vital knowledge and skills in EBP [17, 18]. A 2022 study that conducted a scoping review of EBP implementation in China's healthcare field noted lack of knowledge and skills as critical factors impeding EBP implementation in China [19].

Moreover, implementation theory plays a critical role in guiding EBP in healthcare [20]. However, studies have also highlighted that nurses rarely use theoretical frameworks in evidence implementation projects [21, 22]. A study published in 2018 conducted a bibliometric analysis of the literature related to EBP and found a need for a theoretical framework to support the process of evidence implementation projects in China [23]. In 2020, a scoping review analysing evidence implementation studies in Chinese nursing noted that of the 152 studies it included, 56.58% (86/152) of the literature did not evaluate the quality of the evidence included, and 25.66% (39/152) did not use a theoretical framework to guide the implementation of evidence translation projects [24]. Research has found that the reasons affecting the use of theoretical frameworks by nurses are not only the difficulty of selecting an appropriate theoretical framework from the many theories available in the field [25] but also may be related to the fact that a high proportion of clinical staff are unfamiliar with theories of implementation and behaviour change [26]. For instance, it has been noted that in Australia, the majority of medical, nursing and related health professionals who were successful in applying for the Australian National Health and Medical Research Council Translating Research into Practice Fellows lacked experience in using theoretical frameworks to guide the implementation of evidence [27]. Factors affecting the implementation of evidence, in addition to individual nurses and healthcare organisational factors such as nurses' lack of knowledge of evidence-based nursing, inadequate literature searching skills, busy clinical workloads and lack of support from organisational leadership [28–30]; barriers also include a lack of guidance from the EBP tutor and a lack of collaboration with academic institutions [18, 31], as most evidence-based nursing institutions have close links with universities rather than hospitals [32].

Furthermore, many nursing students with master's degrees in China tend to enter academic institutions rather than clinical organisations [33], and nursing students in China are more inclined to enter universities as academics and researchers after obtaining their doctoral degrees than to enter hospitals as clinical practice specialists [32], making it difficult for clinical nurses to receive expert guidance [18]. Meanwhile, most academic institutions in China are eager to disseminate and implement evidence [32]. However, it has been suggested that the lack of conversation between academic and clinical institutions and the lack of soft skills among researchers to carry out knowledge translation are crucial factors that prevent academic institutions in developing countries from implementing knowledge translation [34]. Meanwhile, an empirical study noted that having a critical mass of EBP mentors in healthcare organisations can increase the confidence of clinical staff to implement evidence, promote evidence implementation and foster an evidence-based culture in hospitals [35]. As such, this study conducts a multi-site evidence-based implementation project of pre-chemotherapy assessment of cancer patients based on collaboration between academic and clinical institutions to help nurses systematically conduct EBP based on implementation theory. The project was implemented based on the evidence summaries and review criteria in the JBI (Joanna Briggs Institute) EBP database [10]. The objectives of this study were to standardise the content of pre-chemotherapy assessment services for cancer patients in clinical hospitals in line with the best available evidence and to improve nurses' compliance with pre-chemotherapy assessment of cancer patients by conducting a national multi-site evidence implementation project in China. The overall aim of the project was to improve the safety of cancer patients during chemotherapy and reduce the incidence of adverse reactions to chemotherapy.

## Method

### Study design

The multi-site evidence implementation project uses the JBI Model of Evidence-based Healthcare as the theoretical framework, the most commonly used theory model in evidence-based practice implementation in healthcare in China [19], the seven steps of the JBI Evidence Implementation Framework as methods [36], and evidence-based audit and feedback as methodology to promote EBPs regarding the pre-treatment assessment of chemotherapy for cancer patients. Clinical audit, as a quality improvement approach, has been advocated for many years to identify gaps and improve healthcare quality in clinics [37]. The JBI approach to evidence implementation, which is firmly grounded in the audit, feedback, and re-audit process, is successful in small or large-scale evidence implementation projects to change practice in the clinic [38–40], with seven stages as follows:

1. Phase 1: Identify the practice area;
2. Phase 2: Engage change agents;
3. Phase 3: Assess context and readiness to change.
4. Phase 4: Review practice against evidence-based audit criteria.
5. Phase 5: Implement changes to practice using Getting Research into Practice;
6. Phase 6: Re-assess practice;
7. Phase 7: Sustainability plan.

### Participants

Clinical medical institutions at all levels were the subjects of the study. Inclusion criteria: 1) hospitals at all levels that provide chemotherapy services to cancer patients; 2) Participation in the project requires the support of the Director of Nursing of the hospital or the Head of Nursing of the implementing unit, and at least one Director of Nursing or Head of Nursing is the leader of the hospital project implementation team; and 3) informed consent to participate in this study voluntarily. As the aim of this study was to implement the pre-chemotherapy assessment guidelines for cancer patients in clinical hospitals, the study did not estimate the required number of hospitals but instead recruited eligible hospitals to participate as much as possible to achieve the goal of facilitating the adoption of the evidence in the clinical context.

The project team recruited participating hospitals throughout China. The recruitment poster with the registration QR code was launched by the Nanfang Nursing Centre for Evidence-Based Practice: A JBI Centre of Excellence at an EBP conference involving 120 hospitals in China. The recruitment posters were then further disseminated on the internet via social media to recruit hospitals nationwide. Interested hospitals completed the application survey by scanning the QR code posted by the online survey platform (www.wjx.cn).

The survey included the following three sections: 1) background and purpose of this evidence implementation project, 2) statement of informed consent, and 3) basic information about the participating hospitals, including the head of the implementation team at the participating hospitals, hospital level, number of beds, location/department where the evidence will be implemented, and the position and educational background of the project lead. Hospitals interested in participating in the national audit project were asked to self-assess the feasibility and suitability of the EBP project in their hospital. Hospitals were required to obtain support and engage at least one nursing manager at their hospital to act as an implementation team leader as a condition of participation. Hospital recruitment was conducted from 18 May 2021 to 7 June 2021.

### The phases of this project

**Fig. 1:**
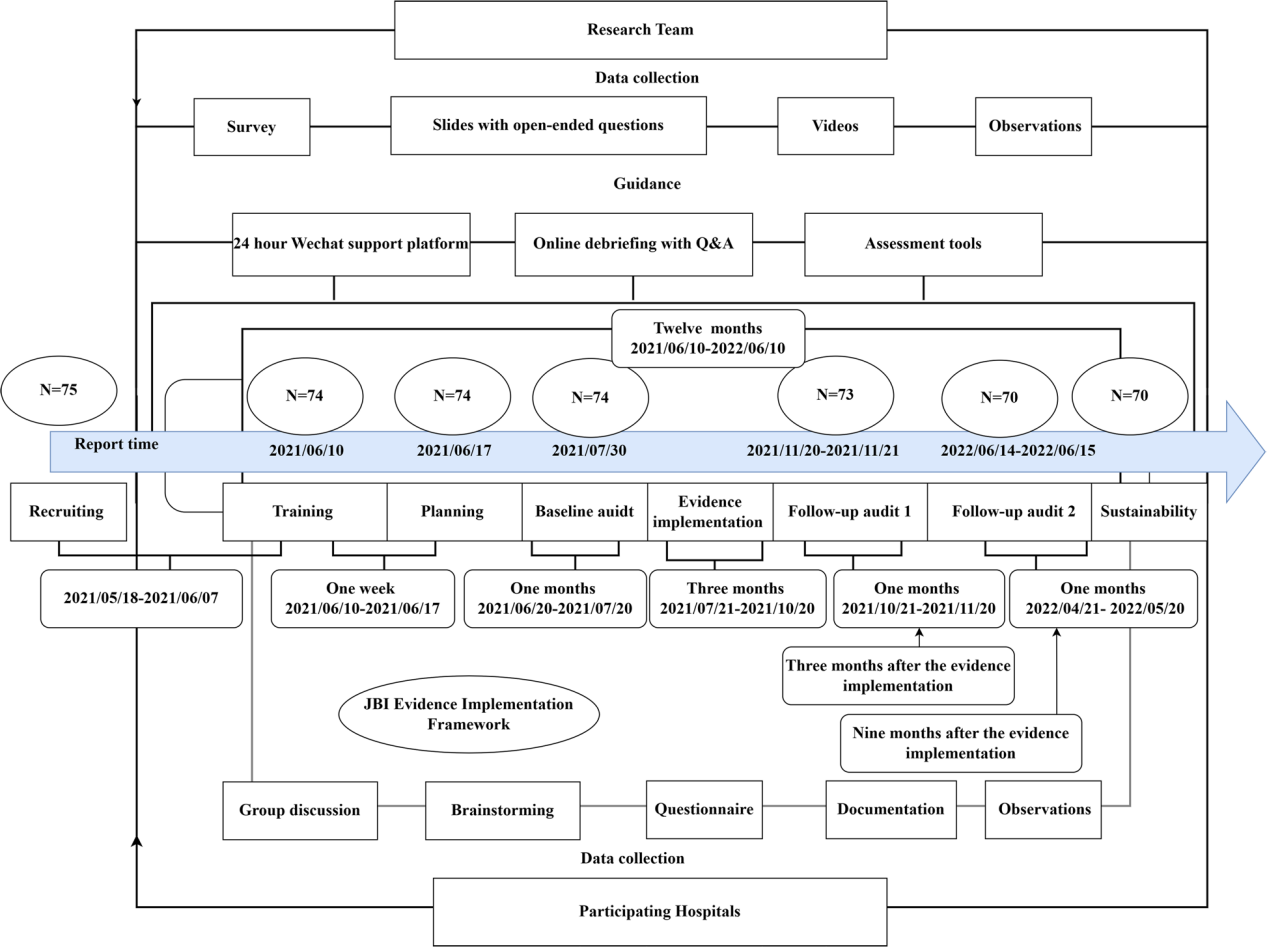
The flow chart for the implementation of the project

In line with the JBI Evidence Implementation Framework [36, 41, 42], this project had seven phases: training, planning, baseline audit, evidence implementation, two rounds of follow-up audits and sustainability assessment. The phases of this project are based on Fig. 1, which shows the flow chart for the project implementation. An online, 24-h social media (WeChat) support platform was provided by the research team. The platform consisted of a JBI-certified evidence implementation trainer from the Nanfang Nursing Centre for Evidence-Based Practice and three full-time postgraduate nursing students studying evidence-based nursing.

Within each site, hospitals used the JBI Practical Application of Clinical Evidence System (JBI PACES) and Getting Research into Practice (GRiP) audit feedback tool for evidence implementation, which consists of three activities [36, 41]:

1. Conduct a baseline audit based on the 12 criteria informed by the evidence. The audit criteria for this project are presented in Table 1 [10], which was developed based on evidence from the JBI evidence summary drawn from research evidence and expert consensus guidelines [43].
2. Analysis of the baseline audit results and implementation strategies to address the barriers between current clinical practice and the best practice recommendations based on the GRiP framework. The GRiP approach aims to compare audit results, identify barriers and facilitators to the use of evidence, and help develop implementation strategies to close the gap between evidence and practice.
3. Conduct two rounds of follow-up audits to assess evidence implementation achievements and identify future practice issues.

**Table 1:** Audit criteria and method used to measure compliance with pre-chemotherapy assessment for cancer patients

Audit criterion	Method used to measure compliance with evidence implementation
1. Nurses have received education regarding the assessment of patients before chemotherapy	Measured by nurses’ self-report This criterion was considered met if nurses reported that he or she has received a formal and comprehensive education program focused on the assessment of patients before chemotherapy and are followed by documented competency assessments
2. The patient’s medical history has been checked	Measured by nursing documents This criterion was considered met if the patient’s medical history has been checked in the nursing documents
3. Presence or absence of allergies has been checked	Measured by nursing documents This criterion was considered met if the patient’s presence or absence of allergies has been checked in the nursing documents
4. The patient’s current diagnosis and cancer status have been checked	Measured by nursing documents This criterion was considered met if the patient’s current diagnosis and cancer status (e.g., recurrence, metastases) has been checked in the nursing documents
5. Recent laboratory results have been checked	Measured by nursing documents This criterion was considered met if the patient’s recent laboratory results have been checked and the abnormal results have been reported to the doctors in the nursing documents
6. The patient’s and/or caregiver’s comprehension of information regarding the disease and treatment plan has been assessed	Measured by nursing documents This criterion was considered met if the patient’s and/or caregiver’s comprehension of information regarding the disease and treatment plan has been assessed in the nursing documents
7. Any previous exposure to chemotherapy agents has been assessed, including previous treatment response and previous toxicities	Measured by nursing documents This criterion was considered met if any previous exposure to chemotherapy agents has been assessed, including previous treatment response and previous toxicities in the nursing documents
8. Physical assessment of the patient has been conducted, including functional status and/or performance status, symptom assessment, and vital signs	Measured by nursing documents This criterion was considered met if: –-vital signs assessment (including temperature, pulse, respiration, and blood pressure) of the patient has been conducted and the abnormal results have been reported to the doctors in the nursing documents –-symptom assessment of the patient has been measured using the MD Anderson Symptom Inventory (MDASI) Core Items and the items that have more than seven scores have been reported to the doctors in the nursing documents
9. Psychosocial assessment of the patient has been conducted and support needs identified	Measured by nursing documents This criterion was considered met if: –-psychosocial assessment of the patient has been measured using the MD Anderson Symptom Inventory (MDASI) Core Items –-for patients that the MDASI single item “sadness” was ≥ 4, the Hospital Anxiety and Depression Scale (HADS), a more detailed screening tool, was used to measure psychological distress. Furthermore, the score of the HADS has been documented in the nursing documents for anxiety and depression separately and has been reported to the doctors in the nursing documents if the score for each scale was ≥ 8
10. The patient’s weight and body surface area have been measured and the impact on chemotherapy dose assessed	Measured by nursing documents This criterion was considered met if the patient’s weight and body surface area have been measured and the impact on chemotherapy dose assessed in the nursing documents
11. Pre-medication requirements have been assessed	Measured by nursing documents This criterion was considered met if pre-medication requirements have been assessed in the nursing documents
12. Assessment of access device required for chemotherapy administration has been conducted	Measured by nursing documents This criterion was considered met if the assessment of the access device required for chemotherapy administration has been conducted in the nursing documents

### Training

The Nanfang Nursing Centre for Evidence-Based Practice conducted online training for all participating hospitals through a half-day online session on 10 June 2021. Each hospital required at least two core members to attend the training. The training included:

- an overview of EBP and the process of the project;
- introduction to the JBI PACES and GRiP audit and feedback tool [39, 44];
- audit methods: describing the results of each audit cycle, interpreting audit criteria of the project and audit compliance methods.

During the training phase, the Nanfang Nursing Centre for Evidence-Based Practice introduced the participating hospitals and briefed them on the background and significance of carrying out the project and the implementation phases of the whole project to deepen the participating hospitals' understanding of the project. Meanwhile, the centre also reminded all participating hospitals of the tasks to be completed at each phase one month in advance in the WeChat group to avoid any omission by the participating hospitals. After training, the centre answered questions from participating hospitals in the project. The nurses from the participating hospitals who received the training were responsible for training other nurses from the implementing site. Meanwhile, the 24hours WeChat support platform provided a way to promptly resolve problems they encountered during the training process.

### Planning

During this stage, each participating hospital site lead formed an EBP team in their hospital, including critical stakeholders, opinion leaders, and clinical leaders. For example, the implementation team could consist of the hospital director of nursing, the head nurse of the implementing unit, the nursing team leader, the head physician of the unit, and the pharmacist in charge. This assures that the project has the support of leaders and opinion champions and facilitates its implementation through a multidisciplinary approach. In addition, during this phase, participating hospitals analyse the specifics of their hospitals and develop evidence-based issues and targets for improvement. By identifying specific problems and setting goals to make it clear to participating hospitals what needs to be improved and what the improvement needs to accomplish. As pre-identifying barriers and developing a package of implementation strategies can help reduce barriers to evidence implementation and promote evidence translation [45], the EBP teams from each participating hospital were asked to anticipate barriers to evidence implementation and strategies to address them based on the JBI GRiP framework within a week using brainstorming and group discussions.

Given the different contexts and cultures of each participating hospital, each hospital also needed to assess whether the methods provided by the research team to measure compliance with best practices could be adopted to their hospital context and culture or whether they needed to be customised. Suppose the participating hospital adapted the audit criteria. In that case, the content and reasons for the adaptation and the relevant sources of evidence must be explained in the relevant sections of the slides.

On the seventh day of the planning phase (17 June 2021), all participating hospitals conducted a live online debriefing session, which included "the formation of each hospital's EBP team", "the methodology used to measure compliance with the 12 audit criteria", and "an analysis of the barriers to implementation of the evidence and proposed strategies to address these barriers". At the end of each hospital's debrief, the online meeting facilitator (the JBI-certified evidence implementation trainer) evaluated and provided feedback on their debrief. If there was any ambiguity in their report, the facilitator asked questions and discussed it further. The briefing lasted 10 h and 20 min.

During the planning phase, three participating hospitals adopted the suggested screening tools in the audit methodology for audit criterion 9 (Psychosocial assessment of the patient has been conducted and support needs identified). One hospital used the Psychological Distress Thermometer [46] as an initial screening tool for the psychological status of cancer patients, and two hospitals used the Self-Rating Scale for Anxiety and Depression [47, 48] and/or Hamilton Anxiety and Depression Scale [49] as a further screening tool for psychological status in cancer patients.

### Baseline audit

Participating hospitals conducted a baseline audit at the practice site against the 12 audit criteria to identify gaps in practice. The inclusion criteria for the audit sample were cancer patients who received chemotherapy during their stay in the hospital and nurses who provide chemotherapy care services to cancer patients at the implementation site. Due to the varied circumstances of the hospitals participating in this project and the aim of this study being to standardise hospitals' pre-chemotherapy assessment of cancer patients, this study allowed hospitals to tailor the audit sample and sampling method to their specific circumstances. For example, for participating hospitals with a large number of cancer patients and sufficient resources, a random sample (random number table method) could be used to collect the sample. On the contrary, for participating hospitals with fewer cancer patients and are poorly resourced, the sample can be collected using continuous sampling (all cancer patients are included in the sample during the audit period until the maximum number is reached). However, to ensure the comparability of the audit project across sites, each hospital's sampling methodology needs to be consistent with that adopted in each audit. In addition, the participating hospitals conducted a small pilot audit at the planning stage to test the feasibility of the audit methodology before the baseline audit started [50, 51]. This baseline audit phase was conducted over one month (20 June 2021 to 20 July 2021).

On 30 July 2021, all hospitals were debriefed online in the manner described above, including but not limited to the methods and results of the baseline audit and the barriers and proposed strategies to evidence implementation. If barriers had changed compared to the planning stage, each hospital was required to highlight the changes in their reporting. After each hospital's debriefing, the online meeting facilitator evaluated and provided feedback. If there was any ambiguity in their report, the facilitator asked questions and discussed it further with hospital teams. After all the hospitals reported, the online meeting facilitator outlined the methodology and requirements for the follow-up audits. The baseline audit online reporting lasted 7 h and 10 min.

### Evidence implementation

After completing the report of the baseline audit, each hospital's EBP team revised the GRiP framework developed during the planning phase following the baseline audit results. Meanwhile, each site moved to the evidence implementation phase. This phase aims to address the gaps between current clinical practice and best practice recommendations based on the baseline audit results, using previously developed implementation strategies. The evidence was implemented in the clinic from 21 July 2021 to 20 October 2021. Two cycles of follow-up audits were conducted three and nine months after the beginning of implementation to review the impacts of implementation of the evidence at each site.

### First cycle of the follow-up audit

Three months after beginning evidence implementation, all sites underwent the first cycle of follow-up audits over one month. To improve the synchronisation of the project, each participating hospital used the same criteria as the baseline for the follow-up audit. All hospitals were required to report the results of the first cycle of the follow-up audit in an online meeting. The same debriefing process was used for all hospitals. This online meeting took place between 20 and 21 November 2021 and lasted 14 h and 40 min. The debriefing content included, but was not limited to, the results of the first follow-up audit and an analysis of the barriers and strategies. If barriers were changed from the baseline audit stage, hospitals must highlight these changes in their reports and develop implementation strategies in response to them. After each participating hospital's presentation, the facilitator answered questions from the participating hospitals and provided feedback on any issues identified during the presentation to ensure consistency in implementing the evidence. Once the reports from the first follow-up audit were completed, the hospitals continued implementing the evidence on-site under the implementation strategy developed.

### Second cycle of the follow-up audit

To further examine the implementation of the project and the impact of the evidence, nine months after beginning evidence implementation, all participating hospitals conducted the second cycle of audits over one month. The same evidence-based audit criteria were used in this process as in the baseline and first-cycle follow-up audit. There were no variations in the types of sampling in each hospital during the process. At this stage, all participating hospitals were asked to report on the project's impact after implementation based on the Donabedian structure-process-outcome (SPO) model [52]. The structure is defined as the setting and administrative processes that guide and assess organisational features that influence practice change; processes are considered integral to care delivery; and outcomes consider patient recovery, return to function and survival [53]. In the second follow-up audit, participating hospitals compared the incidence of chemotherapy adverse events and chemotherapy adverse reactions after the project with those before the project by reviewing nursing documentation. All hospitals were also required to answer an open-ended question, "Is the pre-chemotherapy assessment form for cancer patients embedded in the hospital information system?". Answers were used to assess whether the evidence gets routinely implemented as a norm of nursing practice at the site. The second follow-up audit results were reported in an online meeting, and all hospitals were debriefed as described above. This report was from 14 to 15 June 2022 and took 13 h and 10 min. The session facilitator asked questions and gave feedback for each participating hospital’s report.

### Sustainability

After 12 months of the project’s implementation, all hospitals were required to complete an assessment of the sustainability of the implementation of the evidence in their respective hospitals using the Chinese version of the National Health Service (NHS) Sustainability Model (SM) [54]. The NHS SM was developed by Maher [55] and introduced to China by Jie [54] to help evidence implementation teams identify the sustainability of evidence. Data were collected through an online questionnaire with three sections: 1) an informed consent statement; 2) demographic data including gender, age, title, name and level of the hospital where the project was implemented, name of the project implemented, and role played in the project; and 3) the Chinese version of the NHS SM (Appendix 1: The Chinese version of the NHS SM-English). The questionnaire was distributed via an online survey platform (www.wjx.cn) and completed independently by the project manager or the person with primary responsibility at each site. The NHS SM model comprises ten factors concerning process, staff and organisation. For each factor, the improvement team selects the description that best characterises the improvement project, and the model is scored out of a total of 100 points. A score of less than 55 NHS SM means that the project has a low likelihood of sustainability, and measures need to be taken to maintain the project. Projects with a score of less than 35 require significant effort to address sustainability [56].

### Data collection and quality control

Data collection lasted from June 2021 to June 2022, and slides with open-ended questions, videos, questionnaires, and documentation were used throughout the process. The content included compliance rates for the audit criteria, barriers to implementing the evidence, implementation strategies, clinical impact of the evidence, and an assessment of the project's sustainability. Data collection methods were described in the seven implementation phases of the project.

It is worth noting that the results of this study may be affected by the Hawthorne effect because the implementation teams at the participating hospitals were aware that they were conducting an evidence implementation project [57]. However, this is unavoidable as the purpose of this study was to facilitate the application of evidence in the clinic. Meanwhile, this study has taken some measures to ensure the authenticity and reliability of data collection, as shown below:

1. To ensure the accuracy of data collection, an online meeting was hosted before each audit cycle to further explain the methodology of audit and data collection for all participating hospitals. Four online debriefings (planning, baseline audit, and two cycles of follow-up audits) were conducted by all participating hospitals during the project. All online meetings were recorded via video. Before each presentation, the research team provided a standardised slide template to participating hospitals to guide the reporting for each phase.
2. Two procedures were implemented to collect information to ensure data accuracy. First, before the meeting, all hospitals submitted their debriefing slides and attached documentation to the study team to verify that no information was missed. Second, if the content of a hospital's debriefing during the online meeting did not match the content of the previously submitted slides, or if there was content that was mentioned but not documented on the slides, the meeting organiser followed up with the hospital to clarify. After debriefing, participating hospitals were asked to revise the content of the slides for resubmission to the study team.
3. Numerous methods were used to improve the fidelity of the project. First, the project leader from participating hospitals was required to undertake a field observation of the project and provide a report during the online debriefing, including the nursing documents, such as pre-chemotherapy care assessment forms for cancer patients, admission sheets, as well as photos from the field demonstrating evidence implementation activities. Second, each online debriefing was open to all participating hospitals, including clinical nurses and other healthcare professionals within each hospital. As a result, colleagues who were very familiar with their clinical setting could form oversight of what was reported by each hospital.

### Data analysis and statistics

The data collected from each hospital were extracted independently by two researchers. After the data were extracted, the research team checked the extracted data together to prevent the information from being missed.

The quantitative data were analysed using SPSS version 26.0. Data conforming to a normal distribution are presented as a mean and standard deviation; non-normal variables are reported as median (25% percentile, 75% percentile). The overall compliance rate for each hospital was derived by calculating the average of the compliance rates for all 12 audit criteria. The overall compliance for each audit criterion was calculated by the average of the all-participating hospitals' compliance for each criterion. This study used compliance with audit criteria to reflect the implementation of evidence in participating hospitals. Compliance with audit criteria was calculated as the number of subjects meeting the criteria/total number of subjects audited. A one-way repeated measures ANOVA was used to test the mean difference and 95% CI compared with the baseline audit versus the two follow-up audit cycles. A negative mean difference indicates a decrease in the percentage of compliance, and positive values indicate an increase in the compliance rate. Statistical significance was considered as a *P* value < 0.05. The classification of general hospitals and specialised hospitals in this study is based on the definitions in the National Economic Industry Classification Notes 2017 (online version) published by the National Bureau of Statistics of China [58]. General hospitals include general hospital services, all types of general hospitals and inpatient units of general hospitals (inpatient separated from outpatient) [58]. Specialist hospitals are hospitals that specialise in providing specialist hospital services such as dentistry, ophthalmology, otolaryngology and oncology [58].

Content analysis [59, 60] was used to analyse the extracted data on barriers, strategies, and outcomes of implementing this project. Once the coding of the barriers was completed, the theoretical domain framework (TDF) [61] and Capacity, Opportunity, Motivation-Behaviour (COM-B) model [62] were used to deductively analyse the barrier coding to enhance the reliability of the coding. The taxonomy of implementation strategies of the Cochrane Effective Practice and Organisation of Care (EPOC) [63] was used to classify implementation strategies. The SPO model was used to summarise the impact of the project after implementation. The process of content analysis is shown below:

1. Familiarisation with the text: familiarisation and immersion in the qualitative data was achieved through repeated readings.
2. Generate codes: Codes were extracted from the qualitative data in a word-by-word, phrase-by-phrase, sentence-by-sentence, and paragraph-by-paragraph manner, and the codes were named in a gerund manner to reflect the meaning of the codes, and there was no overlap between the codes.
3. Formation of categories: Codes were categorised and formed into potential categories, and all qualitative data associated with each category were retrieved and categorised into categories. If codes were related to different categories, they were categorised into the most relevant category.
4. Checking of categories: Checking for overlap between categories and for good reflection of coded meanings. Qualitative data were analysed inductively to ensure that no qualitative data were omitted in the above steps, that categories were not lost, and that no new categories were identified in the process of inductively analysing the data.
5. Defining and naming categories: Through deductive analysis, categories were defined and further refined, and the data was analyzed for conformity with the categories. By "defining and refining the categories", the "essence" of each category was identified, and it was determined what aspects of the data were reflected in each category. This study names and defines categories based on the TDF, COM-B model, EPOC implementation strategy taxonomy, and SPO model.
6. Writing the report: when you have a complete set of categories, start the final analysis and write the report. To systematise the links between the themes in the study, we categorised the barriers according to the COM-B model, the implementation strategies using the EPOC implementation framework, and the clinical impact of the evidence using the SPO model. To present the results in an orderly structure, all categories were organised according to how frequently they are mentioned and used by the participating hospitals.

All codes and categories were analysed independently by two researchers. The analysis was reviewed by the research team after completion, consisting of a JBI-certified evidence implementation trainer and three postgraduate nursing students, all with experience in content analysis. The group discussion method is used to resolve discrepancies between raters, and any discrepancies are discussed by the group to reach a consensus. Codes that could not be classified were re-coded and assigned to the most relevant category.

## Results

### Overview of participating hospitals

A total of 75 hospitals applied to join the project, and one hospital withdrew from the project due to a merger of hospital departments before the project was launched. Hence, 74 hospitals from 32 cities in China participated in the national EBP project and finished the training stage. The characteristics of the participating hospitals are shown in Table 2.

**Table 2:** Characteristics of participating hospitals (*N* = 74)

Items	N	%
Change leaders have systematically studied evidence-based nursing	34	45.95
EBP has been implemented in hospitals before	12	16.22
Type of hospital		0.00
General hospitals	12	16.22
Specialized hospital	62	83.78
Level of hospital		0.00
Secondary hospital	6	8.11
Tertiary hospital	68	91.89
Number of departments implemented		0.00
Single department	62	83.78
Multiple departments	12	16.22
Beds per hospital		0.00
0–1,000	23	31.08
1,001–2,000	32	43.24
2,001–3,000	14	18.92
3,001–4,000	3	4.05
4,001–5,000	1	1.35
5,001–6,000	1	1.35
City of hospital		
Guangzhou	15	20.27
Shenzhen	9	12.16
Dongguan	6	8.11
Zhongshan	4	5.41
Foshan	3	4.05
Jiangmen	3	4.05
Shantou	2	2.70
Qingyuan	2	2.70
Zhanjiang	2	2.70
Nanning	2	2.70
Zhengzhou	2	2.70
Changsha	2	2.70
Xi’an	2	2.70
Hangzhou	2	2.70
Jieyang	1	1.35
Meizhou	1	1.35
Shaoguan	1	1.35
Zhaoqing	1	1.35
Zhuhai	1	1.35
Maoming	1	1.35
Yangjiang	1	1.35
Baise	1	1.35
Luoyang	1	1.35
Weihui	1	1.35
Jingzhou	1	1.35
Qianjiang	1	1.35
Nanchang	1	1.35
Chengdu	1	1.35
Dazhou	1	1.35
Kunming	1	1.35
Xiushan	1	1.35
Jinan	1	1.35

### Hospitals’ tailoring of audit criteria and assessment methods

During the planning phase, 74 hospitals tailored the audit approach to suit the specific context of the hospital where they worked. All hospitals used the audit criteria provided by the centre without tailoring. The three participating hospitals adapted the audit methodology for audit criterion 9 (Psychosocial assessment of the patient has been conducted and support needs identified): patients were initially screened for psychosocial status using the MD Anderson Symptom Inventory (MDASI), item: Sadness. If the Sadness score of MDASI was ≥ 4, the patient's psychosocial status was further assessed using the Hospital Anxiety and Depression Scale (HADS). Anxiety and depression scores on the HADS were scored separately and recorded in the nursing documentation. If the patient's anxiety or depression score is eight or more, the nurse is required to report this to the patient's doctor in charge". The reasons why these three participating hospitals adapted the audit methodology for examining the audit criteria are shown below:

1. One hospital did not use the MDASI as an initial screening tool for the psychological status of cancer patients because the participating hospital had previously used the Psychological Distress Thermometer as an initial screening tool for the psychological status of cancer patients receiving chemotherapy. Participating hospitals reported that the nurses were more accustomed to using the Psychological Distress Thermometer as an initial screening tool for the psychological status of patients. Therefore, the hospital adopted the psychological distress thermometer as the initial screening tool for audit criterion 9.
2. Two hospitals switched to using the Self-Rating Scale for Anxiety and Depression and or the Hamilton Anxiety and Depression Scale as a screening tool for audit criterion 9 because the cancer patients treated at these two participating hospitals were children. The participating hospitals reported that the use of the Self-Rating Scale for Anxiety and Depression to assess patients ≥ 9 years of age and the Hamilton Anxiety and Depression Scale for patients < 9 years of age was more appropriate for their hospitals than the HADS. Patients too young to understand the HADS scale are more difficult to understand, and children are unable to use the HADS for self-assessment, which requires healthcare professionals to use the Hamilton Anxiety and Depression Scale to evaluate the child's psychological condition using an assessment by others.

### Barriers and strategies to implementing evidence

Data from 74 hospitals on barriers to evidence implementation and strategies to overcome them were analysed.

1. Barriers to implementing evidence

The top three barriers to the implementation were “lack of knowledge related to change”, the second was “heavy workload”, and the equal third were “healthcare staff disagreed with the evidence and “lack skills for change “ (Table 3).

**Table 3:** Barriers to implementing evidence of 74 hospitals

Com-B	Domain	Barriers	Quote from participants’ report	N	%
Capability	Knowledge	Lack of knowledge related to change	Lack of knowledge of nurses to conduct a comprehensive assessment of cancer patients before treatment; The department nurses are young, lack cancer patient chemotherapy specialized knowledge. (H1)	63	85.14
		Patients lack of knowledge	Patients were poorly educated, and unable to speak Mandarin, China's official language, which caused nurses to struggle to understand their dialect, making it difficult for them to complete pre-chemotherapy assessments and psychological assessments, such as the Anderson Scale of Symptoms and the Hospital Anxiety and Depression Scale; Patients are not understanding of the risk of chemotherapeutic extravasation, may refuse to choose safer intravenous infusion devices. (H19)	21	28.38
		Lack evidence-based knowledge	Clinical nurses lack evidence-based nursing knowledge. Such as the concept of evidence-based nursing, methods of implementation, theoretical frameworks, etc. (H20)	7	9.46
		Change leader lack management experience of EBP	The person in charge of the EBP project lacks management experience, They have not previously implemented evidence translation or evidence-based practices. (H29)	1	1.35
	Skills	Lack skills for change	Nurses lack the skills to evaluate chemotherapy patients, as they are not familiar with the psychological assessment scale used in the evaluation, and lack the skills to communicate with patients. (H2)	24	32.43
	Behavioral regulation	Poor self-management awareness	Patients do not realize that they also need to manage their behaviour such as lifestyle, and cooperate with the nurse for evaluation to restore their health. (H5)	1	1.35
Opportunity	Environmental context and resources	Heavy workload	The workload of clinical nursing is heavy, and it is very difficult for nurses to complete daily nursing work, so they have no time to evaluate cancer patients before chemotherapy. (H3) The implementation of EBP in the department has increased the work of nurses, who previously did not need to assess cancer patients before chemotherapy. (H4)	43	58.11
		Insufficient human resources	Only one nurse assigned to the day chemotherapy and inpatient wards; When EBP encountered difficulties, the department did not have an evidence-based nursing expert or team to guide us to overcome the difficulties; Due to uncontrollable emergencies such as the COVID-19 outbreak being scattered or staff changes in the department, nurses were deployed to support the areas where the outbreak had occurred or were transferred to other departments, resulting in the human resources of the department becoming stretched. (H16)	20	27.03
		No appropriate mechanism has been established	There is no specific workflow for assessing cancer patients before chemotherapy, and nurses often forget what they are supposed to do, or do only a partial assessment. There is also no monitoring mechanism to check whether the nurse has completed the pre-chemotherapy evaluation of the cancer patient, and the quality of their evaluation. (H9)	14	18.92
		Insufficient training resources	The hospital did not train nurses on the knowledge of pre-chemotherapy assessment for cancer patients. (H16)	12	16.22
		Lack of supporting facilities	There is no evaluation tool in the department, such as the evaluation form for cancer patients before chemotherapy. Even if there is such a form, it has not been placed in the e-office system of nurses. (H31) The hospital has no psychiatric unit to treat patients screened by nurses for mental health problems, such as depression. (H36)	11	14.86
		Change of key staff^a^	“With the change of nurse leaders on the unit, the evidence implementation project team needed to not only re-establish relationships with the new nurse leaders, such as mutual trust, but also to get the nurse leaders to understand and support the change.” (H58)	1	1.35
	Social influence	Healthcare staff disagreed with the evidence	The department leaders did not support the EBP project, and doctors believed that it was meaningless for nurses to evaluate cancer patients before chemotherapy and that nurses didn’t have to evaluate them. (H60) The nurse felt that the doctor had already evaluated the cancer chemotherapy patient and there was no need for the nurse to do the same work again. (H30)	24	32.43
		Patients do not know the details of their disease	In China, patients' families often ask doctors and nurses not to tell patients they have cancer, which affects nurses' assessment of patients before chemotherapy. (H33)	5	6.76
		Lack of communication among healthcare staff ^a^	There is a lack of communication between the nurses and doctors on the unit, which makes it very difficult to co-ordinate the work between them. (H69)	1	1.35
Motivation	Intentions	Lack of awareness of change	Nurses were not aware of the need to assess cancer patients before chemotherapy. For nurses, the pre-chemotherapy assessment of cancer patients is only a task required by the department leader, and they don't have the awareness to care for patients in a new way of working. (H41)	23	31.08
	Emotion	Healthcare staff are resistant	The people involved in the change are reluctant to change their previous ways of working and are reluctant to join in; the people involved in the change are resistant to the new. (H66)	16	21.62
		Negative emotions of the patient	Patients have developed a negative psychology due to their illness, such as a sense of stigma, concerns about scales to measure their psychology, fear of being found out to have psychological problems and reluctance to cooperate with nurses. (H11)	5	6.76
	Optimism	Fear that project will not succeed	There are concerns that due to the COVID-19 pandemic, not enough cancer patients are coming to the hospital for chemotherapy, or too much work has been added to the program, leading to the failure of the EBP project. (H15)	11	14.86
	Belief about consequences	Lack of faith in change	Nurses lack confidence in change, and lack the faith to implement it. (H18)	6	8.11
	Belief about Capability	Nurses not competent the change	Nurses cannot accurately assess patients and cannot complete the pre-chemotherapy assessment of cancer patients. (H24)	4	5.41
	Social/professional identity and role	Nurses consider it not their duty	The nurse believes that assessing cancer patients before chemotherapy is the responsibility of doctors, not nurses. (H10)	1	1.35
	Reinforcement	Lack of rewards and punishments	Nurses felt there was no reward for completing cancer chemotherapy and no penalty for not doing so, so there was no incentive to perform the assessment. (H26)	1	1.35

*COM-B* Capacity, Opportunity, Motivation-Behavior

^a^Indicates a newly identified barrier after the planning stage

(2) Strategies to implement evidence

The top three strategies were: “educational meetings”, “local consensus processes", and “patient-mediated interventions” (Table 4).

**Table 4:** Strategists to implementing evidence of 74 hospitals

EPOC category	EPOC sub-category	Quote from participants’ report	N	%
*Implementation strategies*				
Interventions targeted at healthcare workers	Educational meetings	To strengthen nurses' training in basic and relevant knowledge, including evidence-based nursing, epidemiology, statistics, medical English, and the essentials of pre-chemotherapy assessment of cancer patients. (H72)	68	91.89
	Local consensus processes	To describe to clinical nurses and doctors the significance and benefits of pre-chemotherapy nursing assessment in cancer patients, and to gradually build a consensus among healthcare professionals on the implementation of an evidence implementing projects. (H63)	31	41.89
	Patient-mediated interventions	Before admission to the hospital, patients or their families are provided with relevant knowledge, so that patients or their families have an understanding of the disease, patients are taught to use methods of psychological anxiety and depression, and patients are encouraged to carry out self-assessment and report it to the nurses. (H53)	26	35.14
	Inter-professional education	Hire a psychologist to conduct training and teach nursing staff to use psychological assessment tools (H3)	20	27.03
	Monitoring the performance of the delivery of healthcare	Strengthening the regulatory system for nursing management: supervising nurses' use of the new assessment form and the implementation of the assessment system. (H41)	15	20.27
	Local opinion leaders	Obtain the support of the Nurse Manager and Unit Director for the implementation of the project. (H32)	14	18.92
	Educational materials	Enhancement of disease-related knowledge, production of health education leaflets, WeChat tweets and related videos. (H17)	11	14.86
	Managerial supervision	The nurse in charge is responsible for the monitoring of the implementation of the assessment, the team leader and the head nurse supervise it, and the nursing department inspects the unit. (H26)	10	13.51
	Communities of practice	The hospital has a research and innovation team and an evidence-based practice group with previous experience of completing evidence-based practice and can provide relevant guidance. (H29)	5	6.76
	Educational games	Take scenarios and other means to exercise and evaluate the content of the assessment. (H4)	5	6.76
	Reminders	Add relevant elements of the audit criteria to the admitted patient nursing documentation template so that the pre-chemotherapy assessment becomes a routine element of nursing documentation as a reminder. (H8)	2	2.70
	Clinical incident reporting	Regularly carry out a summary of reports of adverse events arising from chemotherapy drug infusions. (H16)	1	1.35
Interventions targeted at healthcare organisations	Organisational culture	Enhancing advocacy, creating a research climate, and shaping a culture that embraces evidence-based nursing. (H16)	1	1.35
*Delivery Arrangements*				
Who provides care and how the healthcare workforce is managed	Role expansion or task shifting	A full-time nursing staff member is established in the project implementation unit. (H47)	22	29.73
	Staffing models	Two additional graduate student volunteers to assist with baseline audits, evidence implementation and data analysis. (H53)	15	20.27
	Self-management	A patient self-management programme to improve the effectiveness of patient self-management	1	1.35
Coordination of care and management of care processes	Care pathways	Develop specific processes for project delivery and points for physician collaboration. (H11)	23	31.08
	Packages of care	Develop an assessment guide to make the assessment more intuitive and utilise the screening tools provided by the project to make the assessment process easy and accurate. (H67)	20	27.03
	Communication between providers	The nurse took the initiative to communicate with the doctor in charge to understand the patient's treatment plan and disease process. The nurse communicates with the doctor to prescribe chemotherapy one day in advance and the nurse activates the standardised pre-chemotherapy nursing assessment form for oncology patients on the day of the patient's chemotherapy. (H10)	10	13.51
	Teams	Formation of a healthcare management team for cancer patients undergoing chemotherapy and practice of integrated healthcare nursing inspections. (H5)	10	13.51
Information and communication technology	Health information systems	Based on the audit criteria and scales, the assessment form will be embedded into the hospital's electronic information system, thereby streamlining the assessment process and shortening the duration of the assessment. (H11)	15	20.27
	The use of information and communication technology	A web-based questionnaire tool, such as QuestionStar, was used to conduct the assessment, making it easier. (H20)	2	2.70
	Smart home technologies	Use of mobile electronic devices for the recording of assessment results. (H42)	2	2.70
Where care is provided and changes to the healthcare environment	Environment	Provide a favourable environment for the assessment, such as a room dedicated to the assessment	3	4.05
*Financial Arrangements*				
Targeted financial incentives for health professionals and healthcare organisations	Pay for performance – target payments	Clarify job responsibilities and incorporate the achievement of the project implementation rate into the performance appraisal of healthcare workers. (H39)	7	9.46
*Governance Arrangements*				
Authority and accountability for health professionals	Professional liability	Incorporate pre-chemotherapy nursing assessment of cancer patients into the duties of the charge nurse. (H39)	2	2.70
	Professional competence	Nurses need to fully understand the clinical significance of 'pain and distress' and explain it to the patient in plain language. Nurses need to be well informed during assessment to avoid disclosure of the condition. (H20)	2	2.70

*EPOC* Effective practice and organisation of care

### Audit details of participating hospitals

1. Baseline audit stage.

**Fig. 2:**
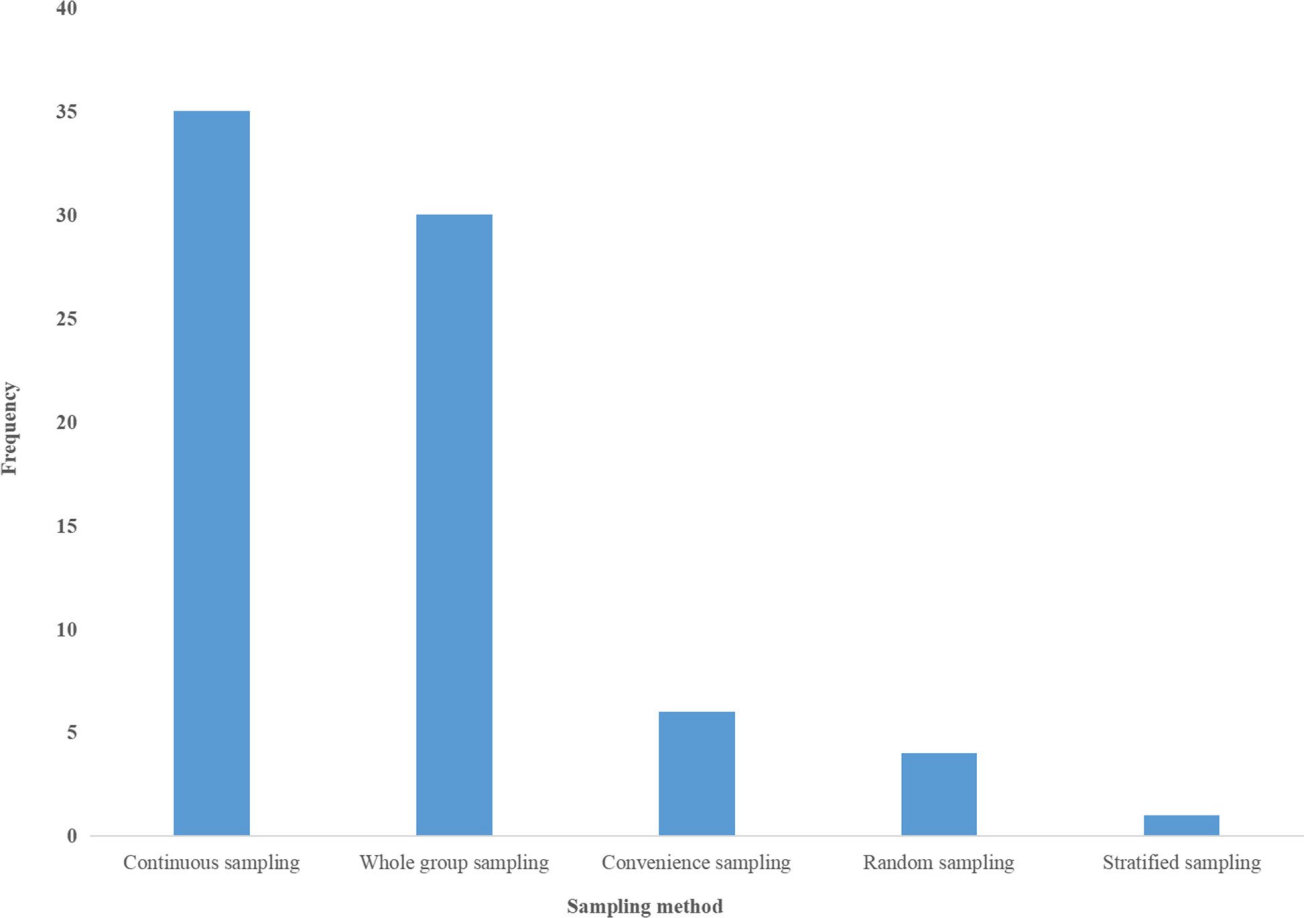
Frequency of sampling methods used (*N* = 76)

Seventy-four hospitals completed baseline audits based on 12 audit criteria, with 1,622 nurses and 2,941 patients reviewed. Of the 74 hospitals, only two used different sampling methods for sampling in two departments, while the rest used only one; thus, the total frequency of the sampling method was 76. More details are shown in Fig. 2.

(2) First follow-up audit stage.

One hospital withdrew before the start of the first follow-up audit due to the change of head nurse in the unit, which was the implementation field. Therefore, 73 hospitals completed the first follow-up audit and audited 1,607 nurses and 2,882 patients through the same methodology as the baseline audit.

(3) Second follow-up audit stage.

Three hospitals withdrew before the second follow-up audit stage due to the lack of human resources and the increase in nurse workload due to the pandemic, and two because of a staff transfer of the project leader. As such, 70 hospitals completed the second follow-up audit and audited 1,594 nurses and 2,813 patients using the same method as the baseline audit.

(4) Overall compliance for each audit criteria.

**Fig. 3:**
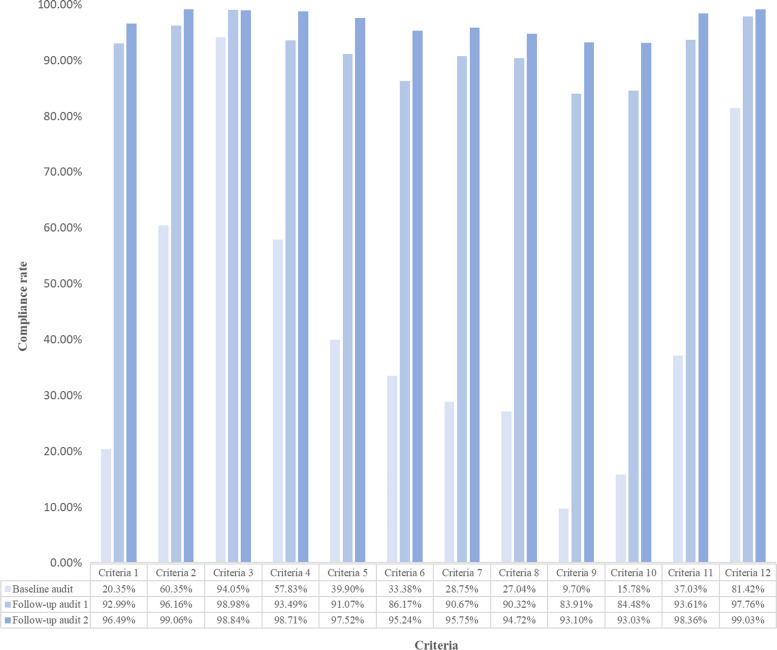
Overall criterion compliance rate for each audit criterion at the three stages. Criteria 1: Nurses have received education regarding the assessment of patients before chemotherapy, Criteria 2: The patient's medical history has been checked, Criteria 3: Presence or absence of allergies has been checked, Criteria 4: The patient's current diagnosis and cancer status have been checked, Criteria 5: Recent laboratory results have been checked, Criteria 6: The patient's and/or caregiver's comprehension of information regarding the disease and treatment plan has been assessed, Criteria 7: Any previous exposure to chemotherapy agents has been assessed, including previous treatment response and previous toxicities, Criteria 8: Physical assessment of the patient has been conducted, including functional status and/or performance status, symptom assessment, and vital signs, Criteria 9: Psychosocial assessment of the patient has been conducted and support needs identified, Criteria 10: The patient’s weight and body surface area have been measured and the impact on chemotherapy dose assessed, Criteria 11: Pre-medication requirements have been assessed, Criteria 12: Assessment of access device required for chemotherapy administration has been conducted

As four hospitals did not complete all audits, compliance rates for 70 of the audited hospitals were included. The individual hospitals' compliance rates for each criterion are shown in Appendix 2: The overall hospital compliance rates for the individual hospitals at the three stages (*N* = 70). The overall criteria compliance for each audit criterion was calculated by the average of the 70 participating hospitals' compliance for each criterion (Fig. 3). The baseline audit noted a gap between clinical practice and the best evidence, with compliance rates below 50% for most of the criteria reviewed. The lowest compliance rate was for criterion 9 at 9.70%. The second follow-up audit noted compliance rates of > 90% for all criteria. The mean of the overall criteria compliance rate for the three audits and the simple effect analysis for the mean overall compliance are shown in Tables 5 and 6.

**Table 5:** Mean overall compliance hospital rate over 70 hospitals and the mean overall criterion compliance rate over 12 criteria

Time	Mean overall hospital compliance rates (Mean ± SD)	Mean overall criterion compliance rate (Mean ± SD)
Baseline audit	42.13 ± 21.31	42.13 ± 26.20
Follow-up audit 1	91.64 ± 9.87	91.63 ± 4.91
Follow-up audit 2	96.65 ± 5.68	96.65 ± 2.27
*F* time	375.548	59.871
*P*	< 0.001	< 0.001

**Table 6:** Simple effect analysis to mean overall compliance rate

Comparison	Overall hospital compliance rate		Overall criterion compliance rate	
	Mean diff	95% CI of diff	Mean diff	95% CI of diff
Follow-up audit 1/Baseline audit	49.50*	(42.95, 56.06)	49.50*	(31.49, 67.52)
Follow-up audit 2/Baseline audit	54.52*	(48.29, 60.76)	54.52*	(34.69, 74.36)
Follow-up audit 2/Follow-up audit 1	5.02*	(2.68, 7.36)	5.02*	(2.61, 7.43)

*: *P* < 0.01

(5) Sustainability of the project.

Seventy hospitals responded to the NHS SM. The median of the total NHS SM score of the 70 hospitals was 87.05 (61.28, 100). Of the 70 hospitals, 55 had NHS SM scores greater than 55, 13 had scores greater than 35 and less than 55, and 2 had less than 35.

(6) Outcomes based on the SPO model.

Seventy hospitals completed all project phases, and the post-implementation impact from the 70 hospitals was analysed with the SPO model. See Table 7 for details of the outcomes.

**Table 7:** The impact of 70 hospitals implementing the evidence

SPO	Categories	Sample quotes	N	%
Structure	Changed nursing processes embedded in hospital work systems	By demonstrating the feasibility and validity of the pre-chemotherapy assessment form for chemotherapy patients at the Chief Nursing Officer's meeting, the Director of Nursing approved the incorporation of the assessment form into the oncology nursing specialty care sheet and into the hospital's electronic information system for the ongoing formalisation of pre-chemotherapy assessment for oncology patients. (H52)	24	34.29
	Changed nursing processes become routine in nursing	Through the implementation of this project, the department has improved the "pre-chemotherapy assessment process for chemotherapy patients" and formed a standard for the department, making it a routine part of the department's daily care. (H22)	7	10.00
Process	Increased patient satisfaction with care services	It has improved patient satisfaction with the nursing service. Since the implementation of the project until now, the department has received several praise flags from patients and many patients' families have expressed their gratitude to the nurses in the department on WeChat for the nursing service we provided for them. (H11)	58	82.86
	Improved knowledge of nurses to implement changed processes	With the help of this project, the hospital project leader has been able to improve the relevant nursing system, standardise the corresponding nursing operation procedures, apply the various assessment forms provided by the centre and develop the corresponding assessment criteria, so that nurses can conduct assessments more easily, quickly and accurately. In addition, through regular training, the nurses' knowledge in conducting nursing assessments for oncology patients has been significantly improved. (H36)	64	91.43
	Nurses' approval of changed process	For the individual nurse, the implementation of the project resulted in a reduction or earlier detection of adverse patient reactions, increased patient comfort and satisfaction, greater recognition of the nurse's work by patients and doctors, and an increased sense of professional achievement for the nurse. The nurses all expressed their approval of the improved nursing process and their willingness to continue applying it. (H56)	53	75.71
Outcome	Decreased incidence of adverse reactions to chemotherapy	After the implementation of the project, based on the feedback from the nurses on the patient's chemotherapy medication needs, the doctor will give the medication before chemotherapy according to the patient's condition and the chemotherapy drugs to be used, so as to prevent the patient from having adverse reactions to chemotherapy drugs during and after chemotherapy, which has led to a reduction in the occurrence of adverse reactions to post-chemotherapy drugs in patients. (H14)	46	65.71
	Prevention of adverse nursing events	Before the project, the nurses did not pay much attention to the psychological problems of oncology chemotherapy patients and did not assess them psychologically; there were adverse incidents of chemotherapy patients jumping off buildings in the oncology department every year. After the implementation of the project, the nurses assessed the chemotherapy patients according to the form provided by the centre in a timely manner and screened out two patients who were heavily depressed and had a clear tendency to self-harm. We promptly asked the neurologist to give them medication and increased psychological care by nurses to prevent self-harm; no suicides occurred within one year of the project. (H73) According to our statistics, after the implementation of the project, we promptly prevented 28 incidents that would have caused adverse chemotherapy events and jeopardised the safety of chemotherapy patients, such as doctors missing premedication, patients on highly emetogenic chemotherapy regimens not being prescribed antiemetics, doctors misreading routine blood results, and patients who had changed their chemotherapy regimens but were given the patient's last hospital order by their doctors in training. (H20)	15	21.43
	Reduction in patient admission days	Before the implementation of the project, there were 2 cases in which the doctors missed to assess the laboratory indicators of the patients, and after the patients were admitted to the hospital, it was found that their laboratory indicators did not meet the standards, and the patients could not receive chemotherapy as scheduled, which prolonged their hospital stay. After the implementation of the project, the nurses will assess the patients' laboratory indicators in advance before chemotherapy, and remind the doctors to check the abnormal laboratory indicators in time, so that the chemotherapy patients can receive chemotherapy in time and reduce their hospital stay. (H15)	2	2.86

*SPO* Structure-process-outcome

## Discussion

The purpose of this study was to standardise the content of pre-chemotherapy assessment for cancer patients in hospitals and to improve nurses' compliance with pre-chemotherapy assessment for cancer patients by carrying out a national multi-site evidence implementation project in China to protect the safety of patients during chemotherapy and to reduce the incidence of adverse reactions to chemotherapy in patients. The research team created a step-by-step approach to the project based on the JBI evidence implementation framework [36], which provides a practical framework for integrating the best available evidence into a distinctive clinical setting to improve the quality of care [41]. In this project, 74 hospitals were included, 4 discontinued the project, and 70 completed the project using the JBI approach to evidence implementation [44]. Through the implementation of this project, the compliance rate of nurses performing pre-chemotherapy assessments for chemotherapy patients in 70 hospitals has improved significantly. It has maintained chemotherapy safety for cancer patients, promoting patient recovery and improved patient satisfaction.

There were several challenging aspects to this implementation project. Firstly, given that the 74 hospitals in the study were from different cities and levels of hospitals in China, it was not easy to standardise the implementation of the project. In addition, the implementation leaders at each site had varying knowledge of EBP and experience in implementing EBP. Therefore, during the project's training phase, we trained participants from each hospital site in the theoretical knowledge of EBP and JBI's evidence implementation approach. During the planning stage, the implementation teams of the participating hospitals were required to assess whether the 12 audit standards and audit methodologies should be tailored to suit the implementation context through brainstorming and group discussions with relevant stakeholders and reporting their assessment results. We also had the centre's EBP mentor provide feedback on all hospital debriefs to ensure that the standardisation of evidence implementation was met while fitting the implementation context at each site. Ultimately, the implementation teams at each site agreed on the 12 audit criteria, and 74 hospitals did not tailor the 12 audit criteria. Only three participating hospitals did not use the recommended screening tool for psychological conditions to screen cancer patients based on the audit methodology for audit criterion 9 based on their patient population and prior assessment history. They chose other screening tools that were more appropriate for their implementation context. This suggests that the audit criteria proposed by JBI can be adopted and used in a standardised multi-site evidence implementation project and are adaptive [40]. Additionally, using EBP mentors for evidence implementation coaching can facilitate evidence implementation in the hospital setting [35].

In this study, the COM-B and TDF were used for the deductive analysis of the identified barriers of 74 hospitals. Two of the top three barriers that ranked as obstacles came from the capacity component of the behaviour change wheel: ' lack of knowledge related to change' (knowledge) and 'lack skills to change' (skills). Meanwhile, the EPOC taxonomy was used to classify the implementation strategies of 74 hospitals. The most frequently used strategy was “educational meetings”, followed by “local consensus processes” and “patient interventions”. These findings are broadly consistent with a previous study that conducted a scoping review of EBP implementation in the Chinese healthcare field and identified the barriers to EBP implementation as a lack of knowledge and skills and the most common implementation strategy as education of healthcare providers and patients [19]. Furthermore, our results also align with the findings of a review describing barriers and strategies to implementing evidence in low to middle-income countries, which suggested that the main barriers focused on knowledge and the most frequent use of educational sessions as an implementation strategy [64].

Secondly, of the 12 audit criteria, the average baseline compliance rate was less than 50% for seven criteria (1, 6, 7, 8, 9, 10, and 11), with the lowest being criterion 9 at 9.70%. These data suggest that most hospitals participating in this project needed to improve their practice to conduct comprehensive pre-chemotherapy assessments of cancer patients as recommended by the evidence. This is consistent with the barriers identified earlier. Meanwhile, this finding is broadly consistent with a previous evidence implementation study of pre-chemotherapy assessment of oncology patients in the breast unit of a tertiary care hospital in Guangdong Province, China [10]. This study conducted a baseline audit of nurses and patients in the breast unit using the same audit criteria as in this study and found that the audit had a compliance rate of 0 for all audit indicators except audit standards 3, 4, 5 and 6.

Moreover, the review of 12 audit criteria identified areas for improvement, focusing mainly on nurse education regarding the pre-chemotherapy assessment of cancer patients and patient education, as well as nurse assessment of patients’ physical and psychological well-being. Based on the findings of the baseline audit, sites used the previously identified implementation strategies to facilitate the implementation of the evidence. Compared to the baseline audit, the first follow-up audit (three months after implementation) showed that the implementation of targeted strategies led to a significant improvement (Mean diff.: 49.50, CI: 42.95,56.06, *P* < 0.01) in overall compliance across all hospitals, as well as a substantial increase in compliance for each audit criterion. These data suggest that targeted implementation strategies based on identified barriers to implementation can facilitate the rapid translation of knowledge into clinical practice [65, 66]. Additionally, implementation strategies need to be selected based on different scenarios; however, there are so many implementation strategies that it is difficult to choose [67]. This study used EPOC to classify the implementation strategies chosen by 74 hospitals and identified the most frequently used strategies: "educational meetings", "local consensus processes", and "patient-mediated interventions". This finding may provide a reference for researchers conducting multi-site EBP to target implementation strategies quickly.

The results of the second follow-up audit (Nine months after evidence implementation) showed a slight improvement (Mean diff.: 5.02, CI:2.68, 7.36, *P* < 0.01) compared to the first follow-up audit, whilst none of the audited standards showed a decline in compliance rates. This suggests that the cyclical use of audit and feedback rapidly facilitates the implementation of evidence in clinical sites at an early stage and makes it sustainable [38]. In conclusion, the provision of training and guidance on EBP implementation by research centres to hospitals and the use of audit standards and audit tools provided by JBI can support the implementation of EBP projects in different contexts and thus facilitate the dissemination of evidence. At the same time, pre-identifying barriers and developing an implementation strategy package before clinical implementation of EBP may reduce the barriers to evidence implementation to facilitate smooth implementation [45].

Thirdly, as this multi-site project was carried out during the COVID-19 pandemic, with frequent outbreaks across China, it was challenging to bring all hospitals together for education and training. To overcome this, we used Dingding, an online education and training platform in China, which allowed us to train all hospitals via live streaming and resulted in numerous benefits. For example, each live broadcast could be recorded and saved via Dingding, enabling all hospitals to watch and learn regardless of space and time constraints, maximising the usefulness of education and training. Hospitals could also showcase the results of their implementation and share their experiences and lessons learnt in evidence-based implementation through the live stream, which enabled all hospitals to progress and learn from each other. Feedback and evaluation of the reporting hospital by the session moderator (EBP mentor) during the live broadcast mobilised peer influence [68], as did sharing the webcast debriefing process, where participating hospitals that were lagging in their project implementation could learn and be motivated by other hospitals with higher standards and progress. This finding is in keeping with previous research indicating that positive peer pressure can increase students' willingness to learn and thus improve learning behaviours [69, 70]. More than half of the hospitals in this study reported that nurses involved in implementing the project went from being initially resistant to EBP to accepting and being willing to implement EBP. This may be related to the way the projects were implemented. The implementation methods of this study enable the outcomes and experiences of all participating hospitals to serve as exemplars for other hospitals, promoting a sense of self-efficacy in the implementation of evidence in hospitals and increasing their recognition and confidence in EBP [71, 72]. Upon reflection, the use of a live online platform is a space- and time-effective way to report the results of the various stages of evidence-based practice that could maximise the number of hospitals and nurses participating, observing, and learning from the practices of other hospitals, enhancing their belief and activity in the implementation of EBP.

Finally, to examine the sustainability of evidence across implementation sites beyond the end of the project, the NHS SM and an open-ended question: "Are pre-chemotherapy care sheets for cancer patients embedded in hospital information systems?" were used. The median NHS SM for the 70 hospitals was 87.05 (61.28, 100), indicating that the evidence is more likely to be maintained after the project's lifespan, yet 15 hospitals had scores below 55, indicating that ongoing measures are still required to promote the maintenance of evidence in the clinic. From the 70 hospital responses to the open-ended question, 24 hospitals had embedded the evidence in the electronic hospital information system, and seven units had incorporated it into their daily care routines. This result indicates that further measures are needed to facilitate the translation of evidence into clinical practice. For example, within the organisation, funding, equipment, and human resources are obtained from hospital managers; outside the organisation, support in funding or policy development is obtained from local academies and governments [67, 73]. Thus, through this study, a relevant group standard has been released through the local nursing society: *Standardized nursing assessment of patients prior to cancer chemotherapy (No T/GDNAS 014—2022)* [74], which can further ensure nurses' compliance with the best evidence in clinical practice and thus promote the maintenance of the project.

### Implications on nursing practice

This study successfully standardised the content of pre-chemotherapy assessment for cancer patients in 70 participating hospitals based on JBI's Evidence Implementation Framework and described the implementation process of the project in detail, which provides a reference for EBP practitioners to carry out large-scale multi-site evidence implementation projects in the future. Moreover, a detailed list of barriers and implementation strategies with case examples was extracted from this study through an inductive and deductive analysis of the barriers identified and implementation strategies undertaken by 74 participating hospitals, which can help inspire EBP practitioners to analyse the barriers and implementation strategies of the evidence implementation projects that they have undertaken. Furthermore, this study provided the participating hospitals with an NHS SM to assess the project's sustainability, which was validated by the Nanfang Nursing Centre for Evidence-Based Practice, and the results of the validation have been reported in previous articles [54, 75]. The model can be readily used by the participating hospitals to assess the sustainability of the project and to implement timely interventions based on the results of the model to facilitate the long-term clinical maintenance of the project. The study also published a group standard: *Standardized nursing assessment of patients prior to cancer chemotherapy*, through the Guangdong Provincial Nursing Association, to strengthen further the sustainability of the project from an institutional perspective.

### Limitations

This project had several limitations. Firstly, the participating hospitals in the project were not recruited as a random sample. Therefore, caution is needed when interpreting the data, such as barriers and implementation strategies, which may have been influenced by the content of the project itself and the implementation context. Although this study deductively analysed the categories of barrier factors and implementation strategies using the COM-B model, TDF and EPOC frameworks to strengthen the reliability of the barrier themes and implementation strategies, the inductive analysis of the barrier factors and implementation strategies was based on implementation scenarios and evidence. Therefore, the categories of barriers and implementation strategies found in this study remain limited by implementation contexts and evidence and must be interpreted cautiously.

Secondly, it was limited by the prevalence of COVID-19 in China. For example, the government has restricted the mobility of residents in the COVID-2019 infected areas and has carried out control and coordination work to ensure that the disease would not spread. Some nurses of the participating hospitals have to travel to the infected areas to support nucleic acid testing of residents in the infected areas for timely detection of COVID-2019 patients and arrangement of quarantine, which has resulted in a shortage of staff in the project implementation unit and hampered the implementation of the project. In addition, the outbreak of COVID-2019 would also make it difficult for the research team to visit the participating hospitals to conduct on-site inspections. This was addressed to some extent by the live online debriefing that enabled the sites to receive monitoring from the participating nurses and other participating hospitals to ensure the implementation of the project, as well as the requirement for participating hospitals to submit photographs and hospital documentation during the debriefing to demonstrate their implementation process and results, further ensuring the project's fidelity.

Thirdly, this study aimed to facilitate the application of evidence in the clinic to accelerate evidence dissemination, so the JBI's Evidence Implementation Framework was used in this study. Whilst this approach is generally recognised internationally, it needs to control for other confounding factors and requires a more systematic implementation approach to test variables that affect EBP implementation. For example, due to the study design, this study did not use the quantitative approach of a randomised controlled trial to validate the evidence and the effectiveness of the implementation strategy. Instead, it used a qualitative approach to illustrate this point. Therefore, the validity of the evidence and implementation strategies is primarily derived from the participating hospitals' subjective perceptions, and this component needs to be interpreted with care. At the same time, this study examined changes in compliance with the evidence in participating hospitals using a pre-and post-audit approach. Although the pre- and post-audit approach is widely recognised as one of the quality improvement approaches, its reliability in reflecting changes in compliance needs to reflect the effect of time on outcomes in the same way as the stepped-wedge design and sequential multiple assignment trials. Future research designs such as effect-implementation hybrid studies, stepped-wedge design, sequential multiple assignment trials, interrupted time series, and multiphase optimisation strategy may be considered further to validate the effectiveness of evidence and implementation strategies.

## Conclusion

This project helped 70 hospitals successfully implement evidence using JBI's Evidence Implementation Framework. This study demonstrated that guiding nurses in evidence implementation through collaboration between academic and clinical institutions can facilitate the translation of clinical evidence [76].

Through categorising the evidence-based implementation strategies into EPOC, this research reveals that "educational meetings", "local consensus processes", and "patient interventions" were the most commonly used strategies. This finding may be beneficial for improving implementation strategies for EBP. Meanwhile, with the help of the audit and feedback approach and targeted implementation strategies, nurses in the 70 hospitals showed significant improvements in compliance with the pre-nursing assessment of cancer patients for chemotherapy. In addition, reporting and feedback on each stage of the multi-site project via live online streaming may increase the confidence of participating hospitals and nurses in EBP and enhance their self-efficacy in implementing the project in terms of outcomes.

Finally, the implementation of the results of this study points to the feasibility of academic centres working with hospitals to promote the dissemination of evidence in clinical to accelerate knowledge translation. Therefore, joint implementation of EBP across regions and organisations to improve the quality of care and patient outcomes may be a desirable way to promote evidence in low- and middle-income countries [77].

### Supplementary Information

Supplementary Material 1: Appendix 1. The Chinese version of the NHS Sustainability Model- English.

Supplementary Material 2: Appendix 2. The Overall hospital compliance rates for the individual hospitals at the three stages (*N*=70).

## Data Availability

The data used to support the findings of this study have not been made available because the participants of this study did not agree for their data to be shared publicly.

## Abbreviations

EBP: Evidence-based practice
JBI: Joanna briggs institute
PACES: Practical application of clinical evidence system
GRiP: Getting research into practice
SPO: Structure-process-outcome
NHS: National health service
SM: Sustainability model
TDF: Theoretical domain framework
COM-B: Capacity, opportunity, motivation-behaviour
EPOC: Cochrane effective practice and organisation of care
MDASI: MD anderson symptom inventory
HADS: Hospital anxiety and depression scale
