# Supplementary material for: Pre-treatment assessment of chemotherapy for cancer patients: a multi-site evidence implementation project of 74 hospitals in China
Source: BMC Nurs. 2024 May 11;23:320. doi: 10.1186/s12912-024-01997-8 (PMC11088226; doi:10.1186/s12912-024-01997-8)
Supplement: Supplementary file 2 — Supplementary Material 2: Appendix 2. The Overall hospital compliance rates for the individual hospitals at the three stages (N=70). [file 12912_2024_1997_MOESM2_ESM.docx]

| Appendix 2 The Overall hospital compliance rates for the individual hospitals at the three stages (N=70) | | | |
| --- | --- | --- | --- |
| Hospital | Baseline audit | Follow-up audit 1 | Follow-up audit 2 |
| H01 | 79.49 | 98.25 | 99.79 |
| H02 | 25.68 | 97.02 | 97.50 |
| H03 | 19.72 | 100.00 | 100.00 |
| H04 | 22.71 | 98.75 | 97.08 |
| H05 | 18.11 | 86.02 | 94.79 |
| H06 | 54.58 | 97.08 | 100.00 |
| H08 | 21.90 | 88.56 | 98.81 |
| H09 | 36.90 | 93.89 | 79.92 |
| H10 | 43.52 | 92.13 | 97.69 |
| H11 | 65.88 | 96.03 | 98.48 |
| H12 | 8.33 | 72.50 | 95.83 |
| H13 | 25.28 | 100.00 | 92.68 |
| H14 | 25.00 | 100.00 | 100.00 |
| H15 | 28.32 | 85.48 | 95.01 |
| H16 | 79.63 | 97.92 | 100.00 |
| H17 | 42.30 | 66.67 | 100.00 |
| H18 | 19.44 | 91.67 | 85.73 |
| H20 | 11.50 | 98.65 | 99.48 |
| H21 | 37.09 | 69.44 | 75.74 |
| H22 | 77.08 | 94.07 | 100.00 |
| H23 | 60.00 | 97.50 | 97.22 |
| H24 | 34.72 | 50.56 | 74.72 |
| H25 | 26.22 | 97.76 | 95.49 |
| H26 | 49.17 | 96.67 | 98.81 |
| H27 | 35.69 | 92.50 | 97.50 |
| H28 | 32.78 | 96.44 | 99.58 |
| H29 | 38.89 | 95.28 | 93.61 |
| H30 | 20.28 | 98.33 | 99.72 |
| H31 | 30.63 | 93.96 | 93.69 |
| H32 | 32.20 | 83.94 | 91.44 |
| H33 | 32.70 | 99.17 | 100.00 |
| H34 | 63.75 | 91.25 | 100.00 |
| H35 | 65.63 | 95.00 | 92.11 |
| H36 | 25.56 | 70.61 | 97.78 |
| H37 | 58.33 | 91.67 | 100.00 |
| H38 | 16.39 | 97.78 | 100.00 |
| H39 | 16.67 | 100.00 | 100.00 |
| H40 | 17.16 | 93.75 | 96.30 |
| H41 | 67.48 | 98.61 | 98.53 |
| H43 | 11.67 | 93.77 | 100.00 |
| H44 | 49.67 | 93.33 | 96.67 |
| H45 | 65.83 | 72.69 | 96.57 |
| H46 | 50.83 | 71.25 | 77.50 |
| H47 | 42.64 | 92.05 | 98.89 |
| H48 | 25.00 | 100.00 | 100.00 |
| H49 | 41.67 | 92.39 | 99.11 |
| H50 | 72.79 | 97.65 | 99.58 |
| H51 | 18.25 | 92.95 | 100.00 |
| H52 | 46.67 | 96.11 | 97.78 |
| H53 | 57.61 | 95.50 | 100.00 |
| H54 | 42.75 | 87.00 | 97.25 |
| H55 | 31.47 | 85.00 | 96.67 |
| H56 | 50.28 | 86.53 | 91.81 |
| H57 | 58.33 | 99.31 | 100.00 |
| H58 | 91.67 | 100.00 | 100.00 |
| H59 | 83.25 | 95.65 | 100.00 |
| H60 | 32.83 | 96.67 | 102.95 |
| H61 | 64.50 | 98.50 | 97.67 |
| H62 | 25.46 | 75.00 | 91.67 |
| H63 | 26.39 | 86.87 | 94.47 |
| H64 | 59.58 | 89.97 | 98.75 |
| H65 | 30.21 | 97.08 | 98.54 |
| H66 | 43.71 | 103.97 | 100.00 |
| H67 | 98.88 | 98.02 | 99.40 |
| H69 | 46.11 | 80.56 | 100.00 |
| H70 | 54.05 | 99.51 | 100.00 |
| H71 | 16.67 | 97.08 | 100.00 |
| H72 | 76.34 | 85.98 | 99.24 |
| H73 | 41.67 | 100.00 | 99.58 |
| H74 | 25.76 | 89.17 | 96.67 |
| Note: The overall compliance rate for each hospital was derived by calculating the average of the compliance rates for all 12 audit criteria. | | | |
